# Different models of anthropomorphism across cultures and ontological limits in current frameworks the integrative framework of anthropomorphism

**DOI:** 10.3389/frobt.2022.863319

**Published:** 2022-08-25

**Authors:** Nicolas Spatola, Serena Marchesi, Agnieszka Wykowska

**Affiliations:** ^1^ Istituto Italiano di Tecnologia, Genova, Italy; ^2^ Artimon Perspectives, Paris, France

**Keywords:** anthropomorphism, human-robot interaction, mentalization, cultural differences, animism

## Abstract

Anthropomorphism describes the tendency to ascribe human characteristics to nonhuman agents. Due to the increased interest in social robotics, anthropomorphism has become a core concept of human-robot interaction (HRI) studies. However, the wide use of this concept resulted in an interchangeability of its definition. In the present study, we propose an integrative framework of anthropomorphism (IFA) encompassing three levels: cultural, individual general tendencies, and direct attributions of human-like characteristics to robots. We also acknowledge the Western bias of the state-of-the-art view of anthropomorphism and develop a cross-cultural approach. In two studies, participants from various cultures completed tasks and questionnaires assessing their animism beliefs, individual tendencies to endow robots with mental properties, spirit, and consider them as more or less human. We also evaluated their attributions of mental anthropomorphic characteristics towards robots (i.e., cognition, emotion, intention). Our results demonstrate, in both experiments, that a three-level model (as hypothesized in the IFA) reliably explains the collected data. We found an overall influence of animism (cultural level) on the two lower levels, and an influence of the individual tendencies to mentalize, spiritualize and humanize (individual level) on the attribution of cognition, emotion and intention. In addition, in Experiment 2, the analyses show a more anthropocentric view of the mind for Western than East-Asian participants. As such, Western perception of robots depends more on humanization while East-Asian on mentalization. We further discuss these results in relation to the anthropomorphism literature and argue for the use of integrative cross-cultural model in HRI research.

## Introduction

When facing or interacting with non-human agents, such as robots, people tend to attribute emotions, intentions or cognition to them, a process called *anthropomorphism* (Fisher, 1991; Epley et al., 2007). The modern, colloquial, use of the concept of anthropomorphism can be broadly defined as the act of assigning human characteristics to non-humans. Because of this broad definition and the growing interest in anthropomorphism in social robotics literature, the label “anthropomorphism” is often used to interchangeably discuss various processes such as *mentalization* (Marchesi et al., 2019; Perez-Osorio and Wykowska, 2020) (i.e. perceiving and interpreting behaviours in terms of mental states such as needs, desires, feelings, beliefs, goals, purposes, and reasons), *humanization* (Spatola et al., 2019a; Spatola, 2019; Spatola et al., 2020) (i.e., treating an entity that is not human as if it was a human), *spiritualism* ^1^ (Martínez-Freire, 1998) (i.e. endowing a non-human entity with a spiritual nature). These three processes are related, but distinct. Therefore, the broad use of the concept of anthropomorphism covering all these three processes blurs the differences between the various phenomenon at stake (i.e., mentalization, humanization, spiritualism).

In the present study, we aimed to define and empirically test a new framework: the Integrative Framework of Anthropomorphism (IFA), articulating the relation between anthropomorphism, mentalization, humanization, and spiritualism processes. We will particularly focus on the dimensions of attribution of mental states (emotion, intention, cognition) in the context of HRI. From a general standpoint, we posit that anthropomorphism, in HRI, would be a process of attributing human-like characteristics to non-human agents that depends on more general individual tendencies towards mentalization, humanization and spiritualism^2^.

Furthermore, our objective was to investigate the role of the main cultural/religious/philosophical factors related to anthropomorphism in social robotics literature (Boyer, 1996; Epley et al., 2007). Animism can be defined as the belief that spirits exist in all material things, both living and non-living. Interestingly, animism is related to anthropomorphism as a prior on which individuals interpret the environment (Boyer, 1996). In the present study, we aimed at investigating this link regarding to cultural difference on the concept of anthropomorphism.

### Anthropomorphism and individual tendencies

As humans, we have the first-hand experience of what it is like to be a human (Epley et al., 2004; Epley et al., 2007). Therefore, anthropomorphism, defined as the attribution of human characteristics to non-humans, is an easily accessible strategy to understand behaviour of other entities (Hampel, 1965; Fisher, 1991; Epley et al., 2007). While the factors eliciting anthropomorphism have been extensively investigated (Epley et al., 2007; Epley et al., 2008; Waytz et al., 2010) there is no clear taxonomy of anthropomorphism. According to literature, we may consider two (related) forms of this concept. First, a physical anthropomorphism directly related to the appearance of the observed entity: the more the shape resembles a human, the higher the anthropomorphism (Duffy, 2003; Harrison and Hall, 2010). Second, a mental anthropomorphism that is grounded in attribution of mind to the observed entity (Waytz et al., 2013). In the present paper, we focus on the latter form. Table 1 summarizes conceptualization of anthropomorphism in literature.

**TABLE 1:** Conceptualization of anthropomorphism across literature.

Authors	General process of anthropomorphism
Piaget. (1929)	Anthropomorphism would rely on an egocentric reasoning in childhood
Heider and Simmel (1944)	When objects are moving without any identifiable cause, there is a tendency to interpret the movements as intentional (i.e., anthropomorphic)
Fisher. (1991)	Two ways of anthropomorphism:
	- interpretative anthropomorphism as the attribution of intentions, beliefs and emotions to nonhuman agents based on their behavior
	- imaginative anthropomorphism as the representation of imaginary and fictional characters as human-like
Mithen and Boyer. (1996)	Anthropomorphism results from the interaction between social intelligence, processing social information, and a mechanism processing biological information
Caporael and Heyes (1997)	Anthropomorphism relies on a cognitive default system restrained when alternative explanations appear more suitable to explain or describe nonhuman actions
Caporael and Heyes (1997)	Anthropomorphism relies on interspecies behavior recognition
Guthrie. (1997)	Anthropomorphism relies on a cognitive default system to interpret ambiguous stimulus in the environment as human-like
Epley et al. (2007)	Schemas about humans are used as the basis for explaining other entities, because this knowledge is more accessible and more detailed than knowledge about non-human entities. This process is moderated by three factors
	- Elicited agent knowledge, that is, the amount of prior knowledge held about an object and the extent to which that knowledge is accessible
	- Effectance, that is, the willingness to interact and understand the environment
	- Sociality, that is, the willingness to establish social connections
Urquiza-Haas and Kotrschal. (2015)	Anthropomorphism relies on a non-reflective and a reflective process. The non-reflective process would be automatic and less affected by cultural differences while the reflective process would be more prone to interindividual differences
Dacey. (2017)	Intuitive anthropomorphism, is a heuristic (cognitive bias) used by our unconscious (folk) psychology to understand nonhuman animals
Airenti. (2018)	Anthropomorphism is grounded in interaction. In interaction, a non-human entity assumes a place that generally is attributed to a human interlocutor. This approach is based on four main assumptions
	- Adults under certain circumstances may anthropomorphize entities even if they know that these entities have no mental life
	- Anthropomorphism is situational and does not depend on a specific target
	- There is no consistency among the entities that are anthropomorphized
	- Inter-individual variability in anthropomorphism is a result of affective states rather than of different degrees of knowledge about the target
Spatola and Chaminade (2022)	Anthropomorphism relies on a default social cognition system that could be bypassed by an active process when sufficient cognitive resources are available. This would result in a switch to a physical cognition system favoring target-specific information and, concomitantly, restricting anthropomorphic inferences (more accessible)

#### Tendency towards mentalization

Mentalization is a level of abstraction in which we explain the behavior of an entity in terms of mental states (Leslie, 1987). It has been opposed to mechanical modes of explanation. According to Dennett, when people have to make sense of simple actions (e.g. a ball rolling on the floor) they may explain it based on physical properties (e.g. the ball rolls on the floor because of an incline) (Dennett, 1971; Abu-Akel et al., 2020). However, when they have to make sense of complex actions (e.g. someone waving at another person approaching), they would tend to explain these observed actions with reference to beliefs, thoughts and intents. Although mentalization is primarily related to human-human interactions, evidence suggests that it can also occur in human-robot interaction (HRI) (Banks, 2020; Perez-Osorio and Wykowska, 2020; Banks, 2021). Interestingly, in HRI, individuals differ in the extent they attribute mentalistic/mechanistic properties to understand robot actions (Gray et al., 2007).

As an explanation process, mentalization is linked to attributions of intentions and cognition, while emotions is less relevant (Kozak et al., 2006). Intentions and cognition (compared to emotions) are attributes more directly related to the ability to plan ahead and think about goals of actions before they are carried out, a process at the core of explaining actions (Malle and Pearce, 2001). Another reason why mentalization would mainly relate to intentions and cognition rather than emotions is structural. Indeed, the mechanisms that underlie attributions of intentions and cognition may differ from those that underlie attribution of emotions (Kozak et al., 2006).

#### Humanization tendency

While anthropomorphism refers to the process of attribution of human characteristics, humanization refers to the process of classifying a nonhuman entity under the “human” category (Spatola, 2019). At the core of humanization is the idea that the conceptual distance between the observer and a observed entity may vary on a continuum. This continuum has been first theorized in social psychology as having dehumanization (Haslam, 2006) (or, to some extent, infrahumanization (Viki et al., 2006; Haslam and Loughnan, 2014)) on one extreme, and humanization on the other. The process of dehumanization means that individuals deprive their fellow humans of human characteristics (e.g., warmth, rationality, agency) because they consider them as “lower-humans”. It may happen in various contexts such as highly hierarchical organizations or structures that higher-positioned individuals may consider individuals with a lower rank as parts of, for example, the production pipeline, dehumanizing them as “machines” (i.e., the mechanistic dehumanization). On the contrary, machines (such as robots) might be “humanized”, a phenomenon studies in social robotics fields. Under certain conditions, for example, as a consequence of a social interaction (Spatola et al., 2019b) or manipulation of group membership (Kuchenbrandt et al., 2013), people may consider robots as close to the human category (i.e., their in-group member) (Kuchenbrandt et al., 2013; Spatola et al., 2019b; Spatola, 2019; Spatola et al., 2020).

As a social categorization process, humanization of robots is related, although distinct (Blanz and Aufderheide, 1999), to anthropomorphism. In other words, because we consider an entity as more or less “distant” from the human category on the humanization continuum, we attribute to them more or less human characteristics. This process from anthropomorphism in the sense that it is a social categorization process while anthropomorphism is an attribution process. This difference is crucial to consider because while we cannot de-anthropomorphized humans, we can dehumanize them. Therefore, we could consider humanization of robots as a tendency on which attribution process occurs. Based on Haslam dehumanization framework we acknowledge the importance of 1) emotions (e.g. emotional responsiveness: interpersonal warmth vs. inertness, coldness), 2) intentions (e.g., agency, individuality vs. passivity, fungibility), and 3) cognition (e.g., cognitive openness vs. rigidity). First, some research point toward the importance of emotion in the dehumanization (or infrahumanization) (Gaunt et al., 2002; Demoulin et al., 2004). While some emotions are believed to be experienced not only by humans and non-humans animals (“primary emotions”; e.g., fear), more complex emotions are believed to be experienced uniquely by humans (“secondary emotions”; e.g., regret) (Turner and Ortony, 1992). This division arises as secondary emotions require complex cognitive processing, which is typically ascribed only to humans, while primary emotions constitute automatic responses to salient stimuli (36). Typically the higher the distance between the self (or the in-group) and a fellow, the fewer the attributions of secondary emotions (Leyens et al., 2001; Demoulin et al., 2004; Viki et al., 2006). Recently, this effect has been used to measure the “humanization” of robots (Kuchenbrandt et al., 2013; Spatola and Wudarczyk, 2020). Second, intentions and cognitions, the capacities to set and reflect on goals, act and influence events and beings (Abele and Wojciszke, 2014), are at the core of the mechanistic dehumanization (Haslam, 2006; Haslam and Loughnan, 2014). Dehumanizing targets are often associated with a decrease of intentions and cognitions attributions (Formanowicz et al., 2018). On the contrary, humanizing robots is associated with an increase of intentions and cognitions attributions to robots (Spatola et al., 2019b).

#### Tendency towards spiritualism

Spiritualism refers to the process of attributing a soul or a spirit to an entity, independent of being a human or not. Spiritualism may apply not only to humans, but also plants, rocks, and any artifacts or natural entity (Segal, 2004). Spiritualism depends on the prior observer’s belief in the existence of souls and spirits; such belief can be grounded in religion, culture or individual representation of the world (Bering, 2006). Spiritualism has not been extensively empirically studied in social robotics. As there is no scientific definition of a soul or a spirit, the two concepts may indicate a conscious (rather than inert) subject (Segal, 2004). Soul or spirit can also be associated to a stream of consciousness, that is, the flow of thoughts in mind. In contrast to mentalization (i.e., interpretation of the behavior of an entity in terms of mental properties) spiritualism refers to a more constant construct (i.e. spirit/soul), that may persist beyond death and is part of a general concept of life (Richert and Harris, 2008).

Here, we propose that “spiritualism of machines or objects” assumes that these entities belong to the category of entities having thoughts (cognition), intentions and emotions (Segal, 2004; Richert and Harris, 2008). As we mentioned, attributing a spirit is to relate to an entity as a conscious subject and therefore attributing cognitive capacity and motives to this subject.

### Anthropomorphism and animism, the role of the culture

As mentioned above, anthropomorphism is the process of attribution of human characteristics to nonhumans. It is a phenomenon that can be observed throughout history all around the world (Mithen and Boyer, 1996; Epley et al., 2007). Although the phenomenon seems to span across the world, some authors hypothesized that some cultures could be more prone to anthropomorphism than others, because of their shared values, norms or beliefs (Jensen and Blok, 2013). Cultures could vary on their tendency towards anthropomorphizing robots because of several factors: 1-their populations may vary in their level of familiarity/exposure with robots (MacDorman et al., 2009; Nomura et al., 2011), 2-because of personal experiences within a given population (Epley et al., 2007), 3-the media they are exposed to, and also 4-the technological development of their country (Razavi et al., 2012). Although these factors have an important influence on shaping the tendency to anthropomorphize robots, the main reason might rely in their historical and religious context (Kaplan, 2004; Bartneck et al., 2005a; Bartneck et al., 2007; Epley et al., 2007; MacDorman et al., 2009; Halpern and Katz, 2012; Sundar et al., 2016; Weng et al., 2019). For instance, Japanese culture has mainly been associated with high anthropomorphism because of the animism beliefs intrinsic to the Shinto religion dominant in that country (Yamamoto, 1983; Jensen and Blok, 2013). Animism is the belief in a shared essence which animates living beings, objects and also natural elements (Bird‐David, 1999). In comparison to the concept of spiritualism, animism is not a representation of an individual with a spirit but a representation of the entire world as animated.

Animism and anthropomorphism can also be considered as overlapping (Guthrie et al., 1997). However, we propose that animism refers to the representation of objects and natural phenomena in a general concept of life (Piaget, 1929), while anthropomorphism is an attribution process and is more context dependent (Airenti, 2018). From an anthropological standpoint, animism can be defined as a belief, a representation of the world. In Fisher’s view we could refer to animism as an imaginative process while anthropomorphism might be an interpretative process (Fisher, 1991). The former is an *a priori* representation of non-human entities as spiritual subjects. The latter is an interpretation of a non-human entity’s behavior or appearance through human lens (Boyer, 1996). Second, while anthropomorphism is an anthropocentric concept, animism is a universalist concept and is often misinterpreted. Unlike anthropomorphism, animism does not assume that non-human entities may embed human characteristics, but that human and non-human entities share a common (not necessarily human) essence. The anthropocentric misinterpretation of animism might be because animism tends to be “westernized” in the anthropocentric approach in which the “spirit” is no more a transcending essence but a property of humans (Sone, 2020).

### General hypotheses underlying the integrative framework of anthropomorphism

**FIGURE 1:**
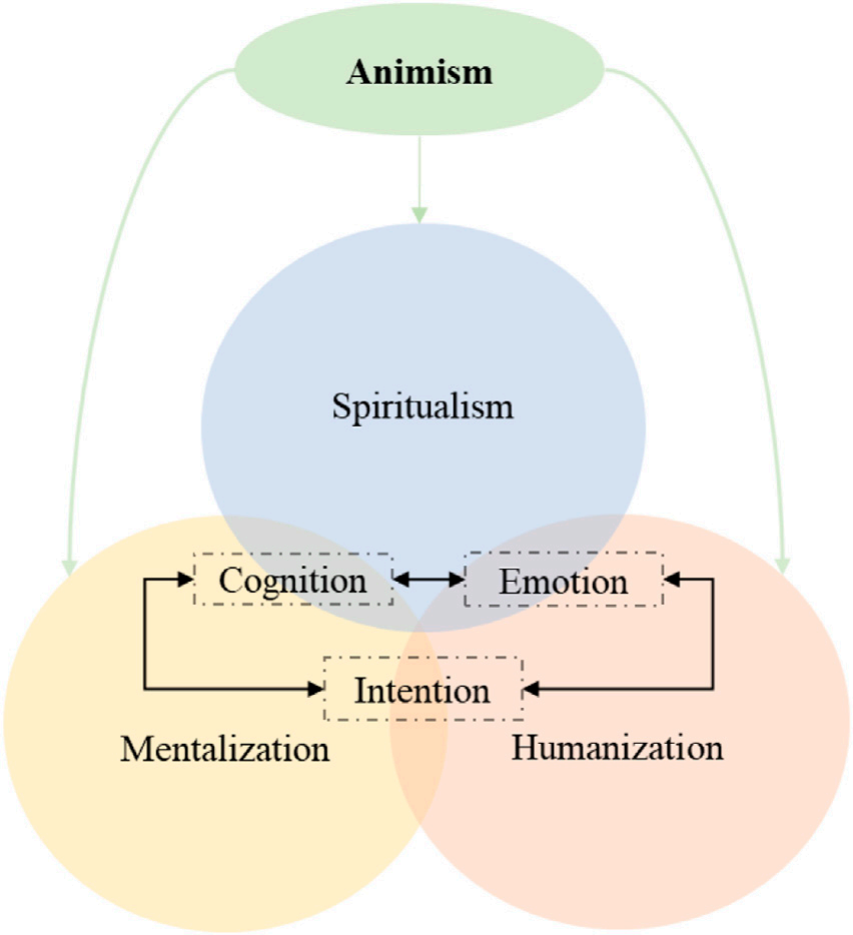
In the IFA, anthropomorphism relates to the attribution of emotion, intention and cognition. These attributions are influenced, by general tendencies such as the mentalization, humanization and spiritualism. These tendencies are mindsets influenced by the cultural context such as animism.

In Figure 1 we present the theoretical IFA that we aim to test using mediation and pathway analyses of data from two experiments.

At the core of the IFA are the attributions of emotions, cognition and intentions. At this level, the core mechanism is the ascription of mind to an entity.

These attributions would depend on (non-exhaustive) processes of mentalization, humanization and spiritualism. At this level, it is important to consider inter-individual differences (further referred to as “tendencies”) which predict the attributions.

Finally, the beliefs/values (culture) would moderate the processes at the individual level. We focus specifically on animism, which we propose to have an indirect influence on attributions through the individual tendencies.

In Experiment 1 we investigated the proposed framework using a multicultural sample. The core idea was to challenge the framework with a heterogeneous sample to evaluate the framework’s reliability and generalizability. Building on Experiment 1, Experiment 2 aimed at comparing the influence of culture as a moderator of the relationship between anthropomorphism and corollary concepts (i.e., mentalization, humanization, spiritualism). The core idea was to test whether the differences in cultural values could moderate the general framework.

## Experiment 1

The first experiment aimed to test the proposed framework of anthropomorphism and the corollary concepts (i.e., IFA) through a pathway model (Figure 1).

### Method

Two hundred and seventy participants took part in this experiment (µ_age_ = 25.85, *σ*_age_ = 5.93, 123 males, 147 females). Participants were recruited on Prolific (see Table 2 for demographic details). All participants received £6.6 as compensation for taking part in the experiment. All participants were naïve to the purpose of this experiment. The sample size was determined based on the desired power (0.80), alpha level (0.05) for mediation models and anticipated halfway (β_a_), hallway (β_b_) paths size (*β* = 0.26) and a τ’ = 0.14. Based on Fritz and MacKinoon (Fritz and MacKinnon, 2007), the minimum required sample size was calculated as 224.

**TABLE 2:** Experiment 1 demographic table.

Country	*n*	Male	Female	µage
Australia	6	3	3	26.5
Austria	1		1	27.0
Belgium	2	1	1	23.0
Cambodia	19	10	9	27.2
Chad	6	3	3	27.5
Czech Republic	1	1		24.0
Swaziland	2		2	30.0
Finland	2	1	1	23.0
France	1		1	40.0
Germany	3	1	2	27.0
Greece	7	4	3	27.3
Hungary	5	1	4	27.4
Ireland	2		2	29.0
Italy	15	5	10	25.7
Japan	2	2		30.0
Latvia	3	3		27.0
Mexico	21	11	10	25.1
Nepal	1		1	32.0
Netherlands	1		1	26.0
Paraguay	34	14	20	23.1
Peru	44	24	20	23.8
Somalia	18	9	9	24.6
South Korea	3	2	1	22.0
Suriname	1		1	37.0
Sweden	1		1	22.0
United Arab Emirates	40	18	22	28.7
United States of America	29	10	19	26.5

The study was approved by the Comitato Etico Regione Liguria and was conducted in accordance with the Code of Ethics of the World Medical Association (Declaration of Helsinki). Each participant provided informed consent before taking part in the experiment by clicking on the “accept” button at the beginning of the survey.

#### Tendency towards animism

Participants completed the Animism Scale for Adults (ASA) (Ikeuchi, 2010). This scale measures the animism beliefs of individuals. Building on Chikaraishi and others’ study (Chikaraishi et al., 2017), we used 2 items of the scale that focus on the attribution of a spirit to non-humans (e.g., I can accept that a sea God lives in the sea and a mountain God lives in the mountain).

In our experiment we replaced the word “God” for “Spirit” as the term “God” may not be adapted to culture with animist or buddhist tradition (which can involve many gods or none) (Pyysiäinen, 2003) or, among others, agnostic and atheist participants (who do not consider the concept of a god) (Sherkat, 2008). This adapted scale proved to have an excellent reliability score (*α* = 0.93).

For each item participants had to indicate the extent to which they agree or disagree with the statement from 1 “Disagree strongly” to 7 “Agree strongly”.

#### Mentalization, spiritualism and humanization measures

**FIGURE 2:**
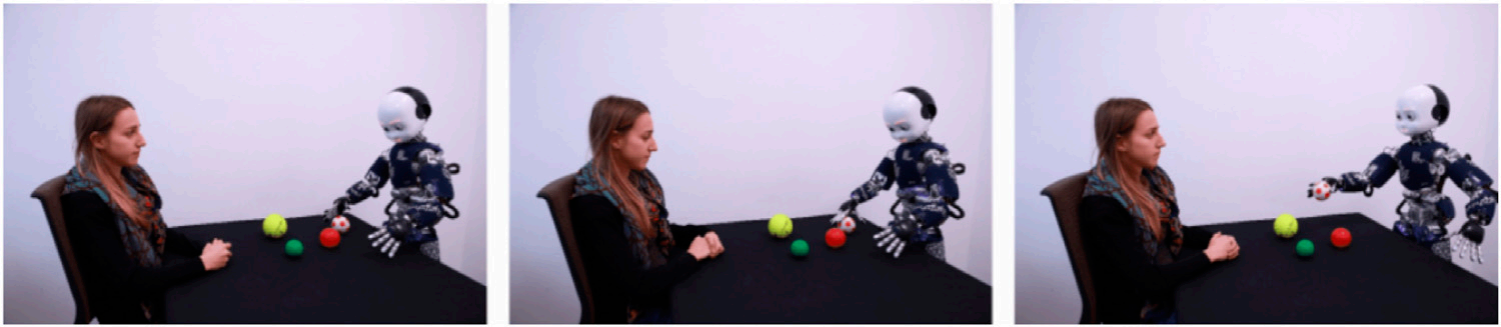
Instance Task scenario.

To measure the tendency to mentalize, participants had to complete the 13 items of the Instance Task (IST) which depicted the humanoid robot iCub in daily activities (Marchesi et al., 2019; Spatola et al., 2021a). Each item of IST was composed of a scenario (Figure 2) and two sentences: one mechanistic (e.g., iCub is scanning the environment) and one mentalistic (e.g., iCub is interested in these objects.).

In the initial IST, participants are instructed to move a slider on a bipolar scale toward the sentence that they consider a more plausible description of the story depicted in the scenario. In the present study, we used the mechanistic [*ω* = 0.75, CI_95%_ (0.71, 0.79)] and mentalistic [*ω* = 0.88, CI_95%_ (0.85, 0.90)] descriptions separately. Participants evaluated separately to what extent each of the mentalistic and mechanistic sentences accurately described the scenario (the presentation order was counterbalanced across trials) from “not at all” to “totally”. This version of the measurement makes it possible to compare mechanistic and mentalistic scores (acknowledging that they are not fully mutually exclusive) and to compute a tendency towards mentalization as the difference between mentalistic and mechanistic scores (which is not possible with the original version).

To measure the tendency towards spiritualism, for each item, participants also had to indicate to what extent they would consider the robot present on the scenario having a spirit/conscious [*ω* = 0.97, CI_95%_ (0.96, 0.97)] from “not at all” to “totally”.

**FIGURE 3:**
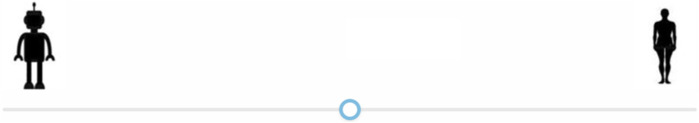
Humanization response silhouettes.

To measure the humanization tendency, grounded in (Spatola et al., 2021b), for each item, participants were explicitly instructed to move the slider on a bipolar scale, made of a robot and a human silhouette (Figure 3) on each extreme of the scale. The cursor was supposed to be moved towards the silhouette that, according to the participants, represented best the degree of human-likeness of the depicted robot action [*ω* = 0.95, CI_95%_ (0.94, 0.96)]. We use this bi-dimensional format as the representation of the (de) humanization continuum with the mechanical and the human pictures at each extreme of the scale.

The mentalization, spiritualism and humanization measures were presented in a random order at each trial (with each IST scenario).

#### Intention, emotion and cognition attribution measures

After the IST scenarios, participants also completed a Mind Attribution Scale to measure the degree to which a participant felt the robot in the scenarios was capable of acting with intention [intention dimension, *ω* = 0.71, CI_95%_ (0.65, 0.77)], engaging in higher order thought [cognition dimension, *ω* = 0.76, CI_95%_ (0.71, 0.80)] and experiencing emotions [emotion dimension, *ω* = 0.93, CI_95%_ (0.92, 0.94)]. Participants made ratings on 7-point Likert-type scales, ranging from 1 (strongly agree) to 7 (strongly disagree). The scale is designed to assess a perceiver’s attributions of intentionality, cognition, and emotions.

#### Control variables

As we aimed to compare various cultures, we measured also the cultural values of participants to control for covariance with the variables of interest. At the beginning of the experiment, participants completed the Cultural Values Scale (CVSCALE) (Yoo et al., 2011). The CVSCALE is a 26-item five-dimensional scale measuring individual cultural values according to Hofstede’s cultural framework at the individual level. The five dimensions are power distance [6 items, e.g., “People in higher positions should make most decisions without consulting people in lower positions”; *ω* = 0.82, CI_95%_ (0.78, 0.85)], uncertainty avoidance [5 items, e.g., “It is important to closely follow instructions and procedures”; *ω* = 0.85, CI_95%_ (0.82, 0.88)], collectivism [6 items, e.g., “Individuals should sacrifice their self-interest for the group”; *ω* = 0.84, CI_95%_ (0.81, 0.87)], long-term orientation [6 items, e.g., “Long-term planning is important”; *ω* = 0.74, CI_95%_ (0.69, 0.79)], and masculinity [4 items, e.g., “It is more important for men to have a professional career than it is for women”; *ω* = 0.80, CI_95%_ (0.76, 0.84)]. In addition, to measure the indulgence dimension posited by Hofstede, we developed 5 items [e.g., “Freedom of speech is important”; *ω* = 0.73, CI_95%_ (0.68, 0.78)]. For each item participants had to indicate the extent to which they agree or disagree with the statement from 1 “Disagree strongly” to 7 “Agree strongly”.

At the end of the experiment, participants had to indicate their country of residence, age and gender.

All the questionnaires are available at https://osf.io/wn4e6/.

## Results

### Data preprocessing

The scores of each dimension for each scale were averaged per participant and standardized. The standardization was a pre-processing step for reliable path model analysis based on regression. We also computed the tendency towards mentalization as the average difference between mentalistic scores and mechanistic scores for each trial from the adapted version of the IST.

### Tendency towards mentalization, humanization and spiritualism

We conducted partial correlation analyses to investigate the relation between the tendency towards mentalization, the spiritualism and humanization variables, taking into account covariance between each variable (and controlling for age and gender of participants). This analysis makes it possible to evaluate the correlation between two variables, taking into account the correlation that both may produce with the third variable. Results showed that tendency towards mentalization, *r* = 0.26, *p* < 0.001, and humanization, *r* = 0.66, *p* < 0.001, were correlated with the tendency towards spiritualism. Also, tendency towards mentalization was correlated with tendency towards humanization, *r* = 0.21, *p* < 0.001.

### Path model

**FIGURE 4:**
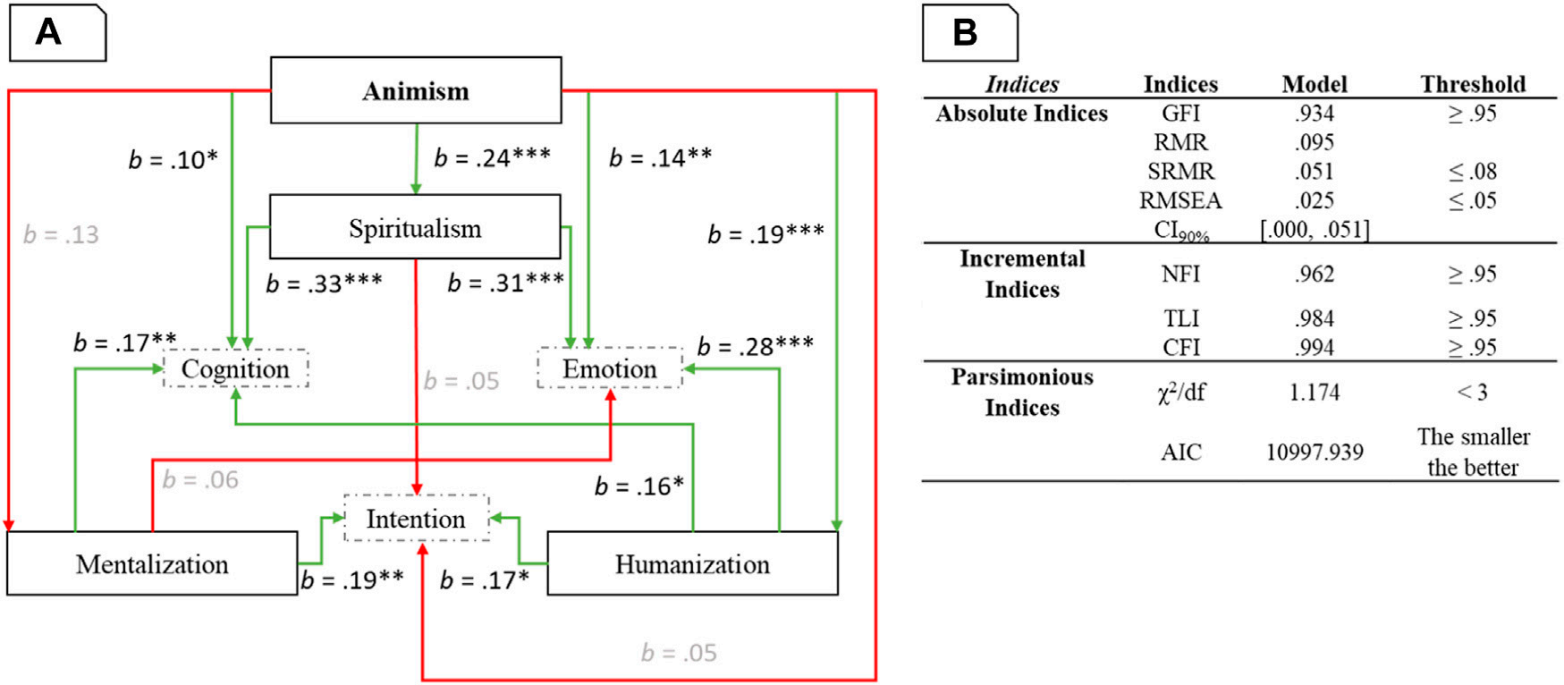
Panel **(A)**. Path model with standardized coefficient. *: *p* < 0.05, **: *p* < 0.01, ***: *p* < 0.001. The non-significant paths are presented in grey. Panel **(B)**. Path model fit indices.

We conducted a path model analysis (an application of structural equation modelling without latent variables). One of the advantages of path analysis is the inclusion of relationships among variables that serve as predictors in one single model. The model (see Figure 4A was estimated in JASP (lavaan) with maximum likelihood estimation method, as the objective was to test a specific model reproducing the covariance matrix of the manifest variables by means the model parameters (Kline, 2015). Figure 4B presents the model fit metrics. We controlled for the significant effects of age, gender, and the 6 cultural values.

The detailed code, analyses and statistics are available at https://osf.io/wn4e6/.

## Discussion

The first experiment aimed at testing a new theoretical framework, the IFA, disambiguating the conceptual relation between core processes related to anthropomorphism (i.e., mentalization, humanization, spiritualism), and those related to mind attribution (three aspects: emotion, intention, cognition). The IFA also included a cultural dimension, namely, animism, as a prior influencing the likelihood to engage in spiritualism.

To test this model, participants evaluated a series of scenarios depicting a humanoid robot in daily activities on various dimensions (mentalization, humanization, spiritualism). These measures were further linked to their attribution of emotions, intentions and cognition to robots in general (as dimensions of the mind) and their animist values.

Results showed a model in which attribution of mind dimensions are related to specific processes (i.e., mentalization, spiritualism, humanization). First, tendency towards mentalization was positively related to the attribution of intention and cognition. Second, tendency towards humanization was positively related to the attribution of emotion, cognition and intention. Third, tendency towards spiritualism was positively related to attribution of cognition and emotion.

Finally, animism beliefs affected spiritualism and humanization (but not mentalization). Contrary to our hypothesis of non-direct influence, animism also directly affected the attribution of emotions and cognition (but not intentions). Overall, the higher the animism beliefs, the higher the spiritualism and humanization tendencies and the attribution of emotion and cognition.

In Experiment 2, we aimed to replicate the results of Experiment 1 and better understand the results.

## Experiment 2

It is often argued that, according to the country of origin, people would be more (or less) likely to anthropomorphize robots. For instance, individuals from East Asian countries (e.g., Korea, Japan) are supposed to have the most positive and anthropomorphic view of robots compared to Western countries (e.g., Germany, United-States) (Kaplan, 2004; Bartneck et al., 2005b; Rau et al., 2010; Lee et al., 2012; Haring et al., 2014; Sone, 2016). To explain this difference, authors proposed that the philosophical animist history of East Asian countries could explain the higher tendency, compared to Wester countries, to endow robots with a mental life (Jensen, 2013; Richardson, 2016). This difference provides a way to test our model in a more hypothesis-oriented approach. This approach is complementary to the more explanatory approach of Experiment 1.”

The second experiment aimed first at replicating and completing the path model of Experiment 1. Second, it also aimed at testing the path model splitting Western and East-Asian countries to disentangle the structural difference in the relationship between anthropomorphism and corollary concepts (i.e., mentalization, humanization, spiritualism) with a different sample type. This approach made it possible to challenge the reliability and generalizability of the model. Third, hypothesizing a difference of animism between East Asian countries (i.e., Korea, Japan) and Western countries (i.e., Germany, United-States), we propose that the East Asian and Western path models should differ on the significant paths. Indeed, while anthropomorphism would be more anthropocentric for Western countries (i.e., humanization), East Asian countries should be less prone to consider the “human” as the reference but the mental life of beings as a shared property (i.e., mentalization, spiritualization). Grounded in previous literature (Kaplan, 2004; Bartneck et al., 2005b; Rau et al., 2010; Lee et al., 2012; Haring et al., 2014; Sone, 2016), for Experiment 2 we recruited participants from East Asian countries (i.e. Korea, Japan) and Western countries (i.e. Germany, United-States). We selected these four countries because these countries have been of primary focus in cross-cultural HRI studies (Kaplan, 2004; Bartneck et al., 2005b; Rau et al., 2010; Lee et al., 2012; Haring et al., 2014; Sone, 2016).

### Method

The method of Experiment 2 was the same as of Experiment 1. The only significant difference was the recruiting of four separate samples (i.e., Korea, Japan, Germany, United-States) gathered in two groups (i.e., East Asian countries, Western countries).

Three hundred and thirteen participants took part in this experiment (µ_age_ = 26.73, *σ*_age_ = 9.35, 94 males, 218 females, 1 non-declared). Participants were recruited on Prolific. All participants received £6.6 as compensation for taking part in the experiment.

Building upon results of Experiment 1, to define the sample size we used Daniel Soper’s sample size calculator for structural equation models (Soper, 2021) based on Westland (Christopher Westland, 2010). With 0.1 anticipated effect size, 0.8 desired statistical power level and *a* = 0.05, the recommended minimum sample size for model structure was 200 (East Asian, *n* = 100, and Western countries, *n* = 100). We extended this minimum to 200 in each country to ensure a sufficient sample size quitting participants who did not fully completed the questionnaire. The demographic details of the participants included in the analyses are presented in Table 3.

**TABLE 3:** Experiment 2 demographic table.

Country	*n*	Male	Female	µage
Korea	99	33	66	26.59
Japan	54	20	34	31.81
Germany^a^	81	17	63	25.06
United States of America	79	24	55	25.14

a One German participants preferred to not declare his/her gender.

#### Tendency towards animism

Participants completed the Animism Scale for Adults (ASA) (Ikeuchi, 2010) (*α* = 0.95).

#### Mentalization, spiritualism, and humanization measures

To measure the tendency towards mentalization, participants had to complete the 13 items of the Instance Task (IST) (Marchesi et al., 2019) standardized by (Spatola et al., 2021a) with the mechanistic [*ω* = 0.70, CI_95%_ (0.65, 0.75)] and mentalistic [*ω* = 0.86, CI_95%_ (0.84, 0.89)] descriptions separated, as in Experiment 1. They also completed the spiritualism tendency [*ω* = 0.97, CI_95%_ (0.96, 0.97)] and the humanization tendency measures [*ω* = 0.94, CI_95%_ (0.93, 0.95)]^3^.

#### Intention, emotion, and cognition measures

As in Experiment 1, participants also completed a Mind Attribution Scale to measure the degree to which a participant felt the robot in the scenarios was capable of acting with intention [intention dimension, *ω* = 0.60, CI_95%_ (0.47, 0.66)], engaging in higher order thought [cognition dimension, *ω* = 0.75, CI_95%_ (0.68, 0.80)] and experiencing emotions [emotion dimension, *ω* = 0.91, CI_95%_ (0.90, 0.93)]. Based on Cronbach’s alpha, the intention dimension was not internally reliable, therefore it will be interpreted with caution.

#### Control variables

At the beginning of the experiment, participants completed the Cultural Values Scale (CVSCALE) (Yoo et al., 2011) with the five dimensions of power distance [*ω* = 0.82, CI_95%_ (0.77 0.85)], uncertainty avoidance [*ω* = 0.86, CI_95%_ (0.83, 0.88)], collectivism [*ω* = 0.84, CI_95%_ (0.81, 0.87)], long-term orientation [*ω* = 0.72, CI_95%_ (0.66, 0.76)], masculinity [*ω* = 0.80, CI_95%_ (0.76, 0.84)] and indulgence [*ω* = 0.72, CI_95%_ (0.66, 0.780)].

At the end of the experiment, participants had to indicate their country of origin, their country of living, their age and gender.

## Results

### Data preprocessing

Similarly to Experiment 1, the scores of each dimension for each scale was averaged and standardized. We also computed the mentalization tendency as the averaged difference between mentalistic scores and mechanistic scores for each trial from the adapted version of the IST.

### Replication of the path model of Experiment 1

**FIGURE 5:**
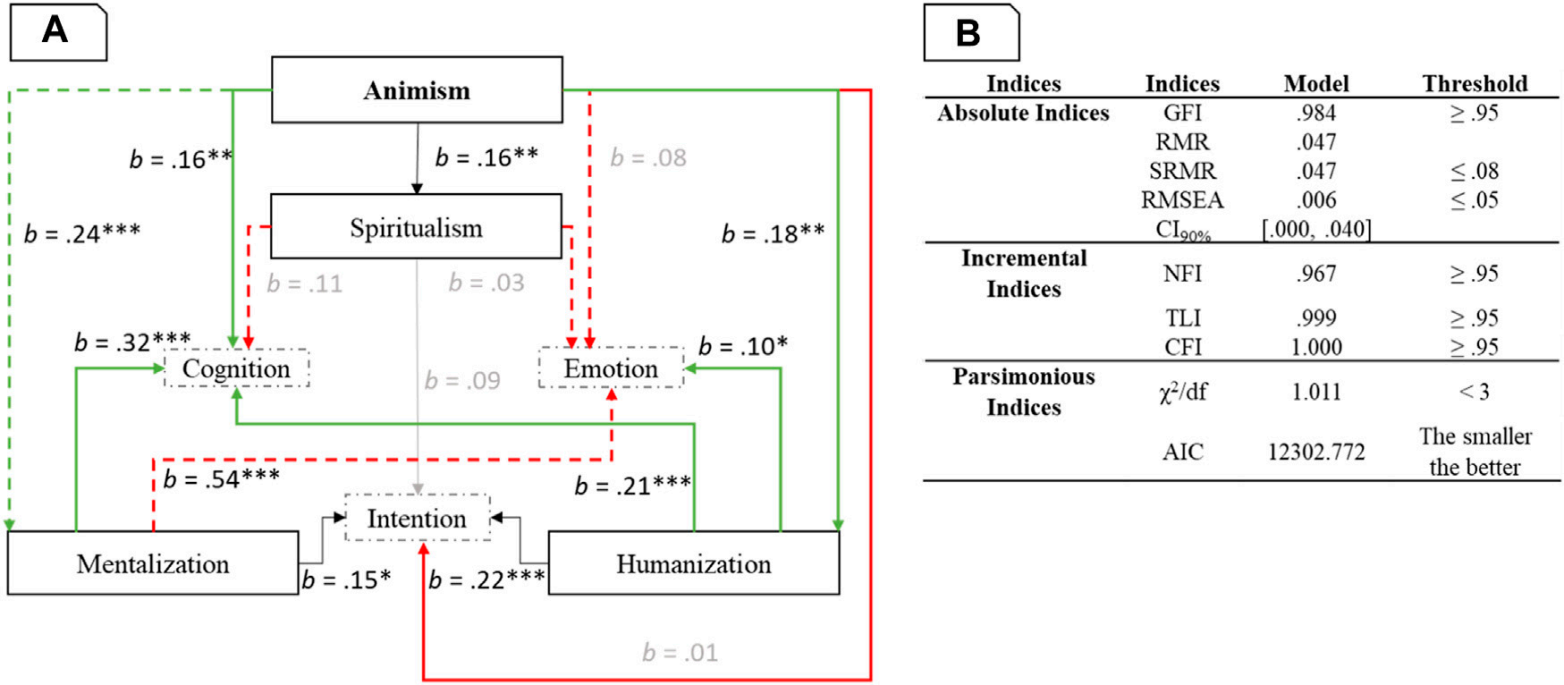
Panel **(A)**. Path model with standardized coefficient. *: *p* < 0.05, **: *p* < 0.01, ***: *p* < 0.001. The non-significant paths are presented in grey. The changing significant paths (compared to model of Experiment 1) are presented in dashed line. Panel **(B)**. Path model fit indices.

We replicated the path model of Experiment 1 in JASP (lavaan) with maximum likelihood estimation method (see Figure 5A,B. presents the model fit metrics for the updated model and the model of Experiment 1.

The detailed code, analyses and statistics are available at https://osf.io/wn4e6/.

The model of Experiment 2 including the overall sample showed some differences with the model of Experiment 1. First, the path between tendency towards spiritualism and attribution of cognition and emotion was not significant. Second, the tendency towards mentalization became a positive significant predictor of emotion attribution. The reason could be the high correlation between scores related to tendencies towards mentalization and spiritualism, *r* = 0.73, *p* < 0.001 (partial correlation). This high correlation could also explain the new (relative to Experiment 1) significant effect of ASA being a positive predictor of tendency towards mentalization.

### East vs. West path model

We first compared the level of animism between East Asian and Western sample with an ANOVA. Results showed that East Asian participants declared higher level of animism compared to Western participants, *F* (1, 311) = 4.40, *p* = 0.037, η^2^ *p* = 0.01.

**FIGURE 6:**
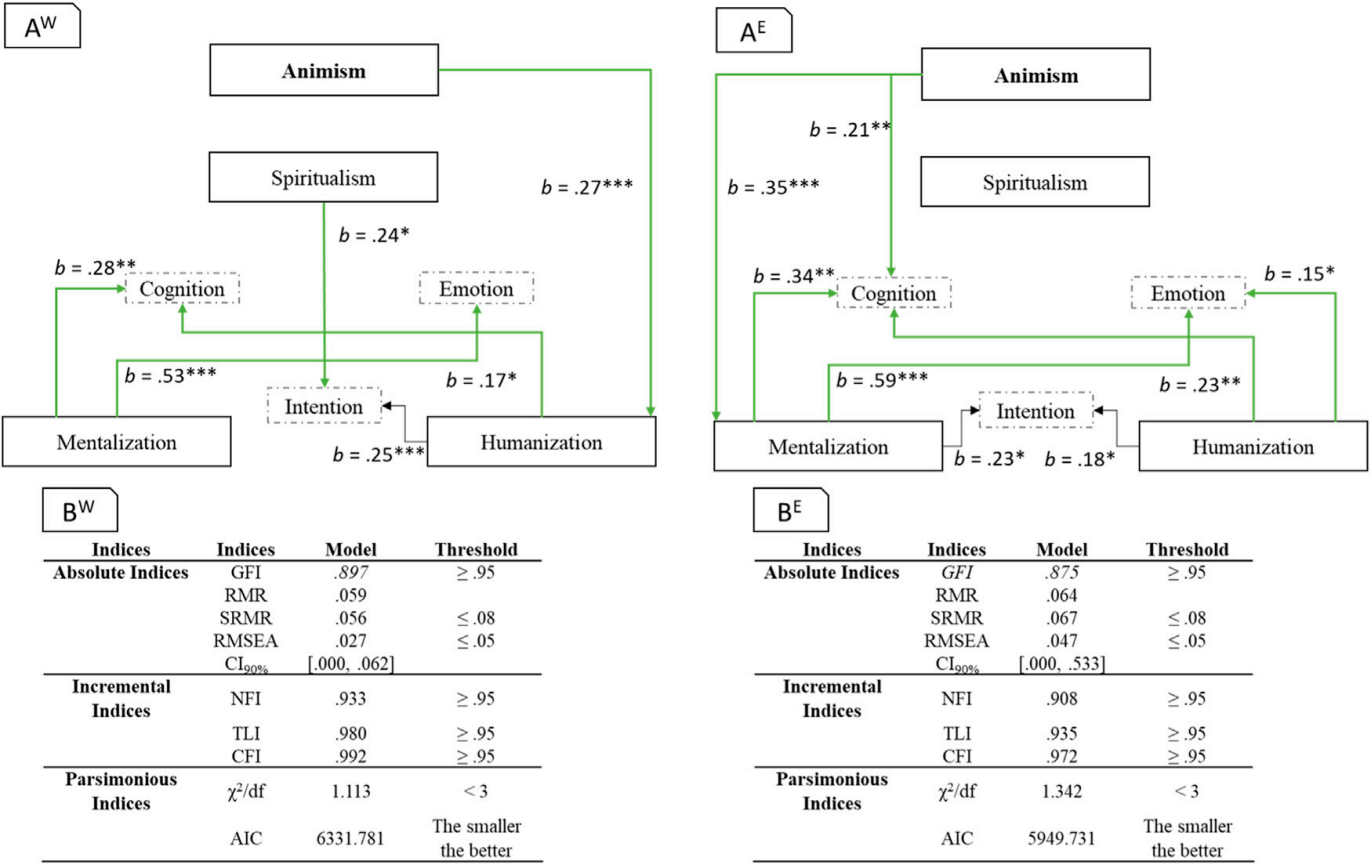
Panel **(A)**. Path model with standardized coefficient. *: *p* < 0.05, **: *p* < 0.01, ***: *p* < 0.001. Only the significant paths are presented with the West sample on the left (A^W^) and the East Asian sample on the right (A^E^). Panel **(B)**. Path model fit indices presented with the West sample on the left (B^W^) and the East Asian sample on the right (B^E^).

We tested the (IFA) model splitting participants according to their country of origin. This resulted in West (United-States and Germany) and East Asian (Japan, Korea) data sets. We then produced a path model for each sample. Figure 6 presents the results.

The detailed code, analyses and statistics are available at https://osf.io/wn4e6/.

The first difference between the West and East Asian model appears to be the path between ASA and the tendencies. While for Western participants, the animist beliefs increased the tendency towards humanization (i.e., considering an agent as conceptually closer to the human group), for East-Asian participants, the animist beliefs increased the tendency towards mentalization (i.e., attribution of mental capacities to an agent). Interestingly, comparing the models, for the Western sample, the *R* ^2^ of tendency towards humanization was 0.129, while the *R* ^2^ for tendency towards mentalization was 0.035. For the East-Asian sample, the *R* ^2^ were 0.002 and 0.119 respectively. Moreover, the effects of mentalization and spiritualism tendencies on intention attribution were reversed across cultures (West vs East-Asian).

## Discussion

Experiment 2 aimed at: 1-replicating the model of Experiment 1 and 2-investigating how the model may vary when comparing Western and East-Asian cultures.

First, the main difference between the model of Experiment 1 and that of Experiment 2 is the path between Spiritualism tendency towards spiritualism and attributions of cognition and emotion attributions, which failed to reach significance in Experiment 2. In addition, animism was now predictive of the tendency towards mentalization, which, in turn, was predictive of emotion.

Second, we found two patterns in the model of Experiment 2 related to the culture of participants (Western vs. East-Asian). Animism was more related to an anthropocentric view (humanization) for Western, relative to East-Asian participants, while, for the latter, animism was more related to a general tendency towards mentalization. It therefore seems that for Western cultures, a spirit is seen as a human characteristic. For East-Asian cultures a spirit is related more to attribution of mental capacities to an agent.

## General discussion

In general, humans tend to assign human mental properties such as intention, emotion or cognition to non-human agents. However, this tendency towards anthropomorphism appears to be ontologically complex. To date, a systemic approach to delineate different concepts underlying anthropomorphism has been missing.

In the IFA, we originally proposed three levels that could be related to anthropomorphism (Figure 1), each level being influenced by superordinate levels. First, animism would be a cultural value shaping a view of the world and containing the underlying two other levels. Second, people would shape their representation of robotic agents based on prior individual general tendencies to attribute mental properties, or by seeing them as more or less distant category in relation to the category of “Humans”. Finally, contextually, they would attribute specific characteristics such as intentions, cognition and emotions to a non-human robot agent.

To test the IFA, we conducted two experiments in which participants from different cultures had to fill a series of questionnaires. In Experiment 1, we aimed to test the IFA with a culturally diverse population. In Experiment 2, we attempted to replicate results of Experiment 1 and compare how the IFA could be moderated by Western vs. East-Asian cultures.

### The cultural level

**FIGURE 7:**
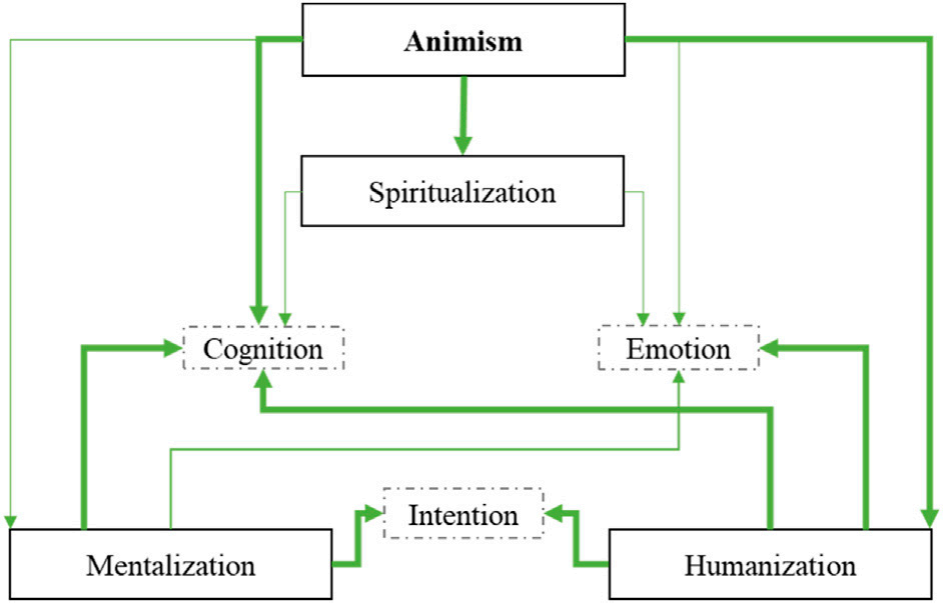
Summary model encompassing pathway model analyses from both Experiment 1 and Experiment 2. The figure only presents the significant paths (all positive). Paths in bold revealed to be significant in both experiments.

Figure 7 summarizes the models of Experiment 1 and 2. Overall, the IFA, embedding three-level, seems to be validated. We indeed found the influence of animism on the mentalization (Experiment 2), spiritualism (Experiment 1 and 2) and humanization (Experiment 1 and 2). The higher the animism beliefs, the higher these tendencies. In line with previous studies (Papadopoulos and Koulouglioti, 2018), in our framework, animism is thus conceptualized as a cultural basis that may increase or decrease the tendencies towards mentalization, humanization and spiritualism at the individual level.

Interestingly, we found two different patterns when modelling the data from the Western and East-Asian samples separately (Experiment 2). For Western participants, animism was related to humanization while for East-Asian participants, animism was related to mentalization. As we hypothesized, Western cultures proved to be more anthropocentric than East-Asian cultures. Humanization is the tendency to represent a robot on the robot-human continuum. On the other hand, mentalization is the tendency to attribute mental capacities to a robot (independent of human reference, as depicted in the model). The difference between cultures in how animism affects anthropomorphism-through either humanization (the West) or mentalization (The East)—illustrates that anthropomorphism might have different (culturally-flavoured) facets. This confirms Urquiza-Haas and Kortschal (Urquiza-Haas and Kotrschal, 2015) theory which highlights the interplay between cultural differences and individual variability as a crucial process in anthropomorphism. In terms of more methodological considerations, these results demonstrate that comparing anthropomorphic tendencies in the various questionnaires or tasks available in HRI literature (Bartneck et al., 2009; Carpinella et al., 2017a; Marchesi et al., 2019; Spatola et al., 2020) may result in misleading interpretation due lack of delineation of constructs. Our results show that concepts may be epistemologically different and attributing mental properties to a robot in an anthropocentric culture is not the same as in a culture with less anthropocentric values. In other words, the question “Does this robot have a mind” in an anthropocentric culture would be closer to a question “To what extent is this robot like a human” while in a non-anthropocentric culture, the same question would be closer to “Can this robot think and have emotions”.

Our results also showed that, when considering the tendencies towards mentalization, humanization and spiritualism, animism directly effected attributions of cognition (Experiment 1 and 2) and emotion (Experiment 1) but not of intention. This is quite interesting, as it further supports the claim that cultural values might affect different aspects of anthropomorphism differently.

### The individual level

In both experiments we found that mentalization, humanization, and spiritualism were parallel tendencies (significant when controlling covariance with other tendencies) and, as such, were separate, but correlated, constructs (Experiment 1). In Experiment 2 with found a high covariance between mentalization and spiritualism making the effect of the spiritualism tendency on the anthropomorphic attributions deplete, which puts into question how to delineate the three tendencies.

From a general viewpoint, these results support two types of processes. The first one is as a process of categorizing a robot on the humanization continuum. It determines if a robot is “like a human” (Spatola and Urbanska, 2019). The closer to the human, the higher the attribution of intentions, cognition and emotions, as those are human characteristics (Spatola et al., 2019b). The second process (partially) independent of the “human-like” categorization, relies on the ascription of a mind in two correlated forms: mentalization and spiritualism. Mentalization is manner of explaining behaviour. Spiritualism is the idea that a robot shares commonalities with other living beings populating the world. This subdivision echoes Fisher’s view of anthropomorphism (Fisher, 1991). Fisher proposed that anthropomorphism could be divided in an interpretative (i.e., situational explanation process) and an imaginative (i.e., general representation) forms. Mentalization would be the interpretative form and Spiritualism would be the imaginative form of mind attribution to non-human agents, such as robots.

Interestingly, Experiment 1 and Experiment 2 showed that the attribution of intention, emotion and cognition to robots could depend on multiple tendencies in parallel, arguing that anthropomorphism is a complex, rather than a unitary, process-not only in terms of motivational factors, as posited by Epley and others (Epley et al., 2007), but also in terms of processes underlying anthropomorphic representation of a non-human agent. For instance, attributing “intentionality” to a robot may result from a social categorization process (humanization) or/and an interpretation process (mentalization). Therefore, in research on anthropomorphism, should take these epistemological distinctions into account.

### Going further

#### The linguistic

“If we consider Fisher’s interpretative anthropomorphism (Fisher, 1991) and the actual tools requiring individuals to evaluate a robot on various scales, we may question their comparability between cultures. Let us consider the Godspeed questionnaire (Bartneck et al., 2008), the Robotic Social Attribute Scale (Carpinella et al., 2017b), or the Human-Robot Interaction Evaluation Scale (Spatola et al., 2021c) that our research team used in different studies. In the Godspeed questionnaire, terms such as “Fake-Natural” or “Artificial-Lifelike” may be associated to very different signifieds (which pertains to the form) between two cultures while the signifiers (which pertains to the content) remain the same. Godspeed example is even more relevant as the evaluation is not only based on a single signified but a continuum between two. Therefore the representation of what means “Artificial”, what means “Lifelike”, and what is the relationship between both is deeply influenced by a prior view of the world influenced by, among others, cultural factors (Thompson et al., 2016). This effect of culture on language and representation of the environment is anchored in evolution of human cognition. Cultural linguistic psychology literature shows effect on basic human concepts such as time and space (de la Fuente et al., 2014). For instance, while Western individuals tend to represent the future as being front of them, Moroccans conceptualize the past as in front of them and the future as behind them due to the direction of writing (de la Fuente et al., 2014). In other words, asking to reflect about the future, a European and a Moroccan would share the signified but not the signifier. As such they will be able to answer question about the “future” but their response will not correspond to the exact same concept. With respect to these results, considering anthropomorphism and related concepts out of these cultural linguistic differences seems at least questionable.

#### A culturally intrinsic issue

For instance, as our results showed, if we consider the concepts of “consciousness”, “human-like”, and “responsive” that are present (or with equivalents) in the questionnaires we mentioned, the signified and the semantic link between the signifiers would diverge between a Western and an East-Asian individuals. The first would consider these concepts through an anthropocentric view while the latter would have a less human centred view. Therefore, if two participants each from a different culture both answer that “responsive” fits totally with the concept of a robot, would it be the same response Can one conclude based on such response that one culture or the other anthropomorphizes robots more Unfortunately, there is no clear answers to these questions.

To address this issue, a possibility could be to translate the items based only on the signifier (Blenkinsopp and Pajouh, 2010; Akbari, 2015). However, the relationship between culture and language, especially in terms of culture-specific items, is among the most thorny issues a translator or interpreter has to deal with and some concepts simply do not exist in some cultures (e.g. šiˑšaˑwiɬtaqyo in Nuu-chah-nulth corresponding to “Powered by a monstrous supernatural porcupine-like creature”). Another solution could be to use cultural values and norms as covariates in analyses and models when evaluating anthropomorphism. Indeed, simplification of cultural concepts, such as anthropomorphism, that are intrinsically multifactorial is, by definition, a dead end as it proceeds from a biased view of the world as we mentioned (Fisher, 1991; Epley et al., 2007).”

### Limitations

As illustrated by Figure 7, while Experiment 1 and Experiment 2 are overall consistent, we observed differences, especially regarding spiritualism. Indeed, spiritualism and mentalization seem intricate concepts that may be difficult to distinguish. This issue is even more critical, considering the variability across cultures regarding the ontology of the concepts of “spirit” and “mind”. For instance, Roazzi and others showed in a cross-cultural study that culture may recruit intuitive foundations, such as essentialism, intuitive psychology, and vitalism differently to define different aspects of immaterial identity (Roazzi et al., 2013). In some cultures, “spirit” might be related to a higher extent to emotion than to cognition or intention. Similarly, “mind” might also be related to different attributions. In our model, this could result in cross-cultural differences regarding the different paths.

Another aspect that needs to be considered in future research is that our statistical model of the IFA is unidirectional - from the cultural to the attributional level. While in this paper, we only present a unidirectional model, we acknowledge that the different levels might influence one another in a more bi-directional fashion. However, one needs to consider that the higher levels might be less influenced by the lower levels: while it is plausible (and shown by our results) that cultural values moderate the level of attributions cognition, emotions and intentions, it is less likely that individual specific attributions moderate general tendencies towards mentalization, spiritualism and humanization, and even less the cultural values such as animism (Schmitt and Blum, 2020).

Finally, the IFA might also be incomplete. For instance, one could propose that individual personality traits play a role in anthropomorphism at various levels, as individual traits proved to be reliable predictors of anthropomorphic attributions in literature (Syrdal et al., 2009; Nomura et al., 2011; Spatola and Wudarczyk, 2020).

## Conclusion

While anthropomorphism is a broadly used concept, its epistemology is still to be discussed and investigated. In two experiments, we demonstrated that anthropomorphism should be, at first, considered in a cultural/individual/attributional context. Paradoxically, from the anthropomorphism definitions we reviewed, only a few consider the cultural dimension and none discusses that anthropomorphism could be considered as various processes according to this cultural dimension. The various processes underlying anthropomorphism should be delineated. For instance, attribution of intentions might be considered as a mentalization process (East cultures), or as a categorization of an agent on the humanization continuum (Western cultures).

More importantly, the present results show that the concepts of anthropomorphism, mentalization, humanization, and spiritualism or animism, as used in the state-of-the-art research, are deeply Westernized and interpreted through the lens of Western cultural representations. It seems therefore necessary to extend research and theoretical frameworks beyond the Western countries. Also, it is important to acknowledge that, even if a concept exists in two cultures, the semantic may highly differ and conduct to misleading interpretation. In the case of anthropomorphism, the.

## Data Availability

The datasets presented in this study can be found in online repositories. The names of the repository/repositories and accession number(s) can be found below: https://osf.io/wn4e6/.

## Footnotes

1 We use the term “spiritualism” and not “animism” to distinguish the process of attribution of spirit to an entity (i.e., spiritualism) from the cultural and religious phenomenon (i.e., animism) which is a general view of the world.

2 We do not consider these three processes as exhaustive factors relating to anthropomorphism.

3 Another practical rational to include two countries per region was the low number of East Asian participants available on Prolific. For instance, we were not able to recruit more Japanese participants as the experiment remained active for 1 week.
